# Saponins from *Allii Macrostemonis Bulbus* attenuate atherosclerosis by inhibiting macrophage foam cell formation and inflammation

**DOI:** 10.1038/s41598-024-61209-w

**Published:** 2024-06-05

**Authors:** Shutian Zhao, Huijun Guo, Liang Qiu, Chao Zhong, Jing Xue, Manman Qin, Yifeng Zhang, Chuanming Xu, Yanfei Xie, Jun Yu

**Affiliations:** 1 Translational Medicine Centre, Jiangxi University of Chinese Medicine, Nanchang, Jiangxi China; 2 Department of Cardiovascular Sciences and Center for Metabolic Disease Research, Lewis Katz School of Medicine, Temple University, Philadelphia, PA USA

**Keywords:** Saponins from *Allii Macrostemonis Bulbus*, Atherosclerosis, Macrophage, Inflammation, Pharmacology, Atherosclerosis

## Abstract

*Allii Macrostemonis Bulbus* (AMB) is a traditional Chinese medicine with medicinal and food homology. AMB has various biological activities, including anti-coagulation, lipid-lowering, anti-tumor, and antioxidant effects. Saponins from *Allium macrostemonis Bulbus* (SAMB), the predominant beneficial compounds, also exhibited lipid-lowering and anti-inflammatory properties. However, the effect of SAMB on atherosclerosis and the underlying mechanisms are still unclear. This study aimed to elucidate the pharmacological impact of SAMB on atherosclerosis. In apolipoprotein E deficiency (ApoE^−/−^) mice with high-fat diet feeding, oral SAMB administration significantly attenuated inflammation and atherosclerosis plaque formation. The in vitro experiments demonstrated that SAMB effectively suppressed oxidized-LDL-induced foam cell formation by down-regulating CD36 expression, thereby inhibiting lipid endocytosis in bone marrow-derived macrophages. Additionally, SAMB effectively blocked LPS-induced inflammatory response in bone marrow-derived macrophages potentially through modulating the NF-κB/NLRP3 pathway. In conclusion, SAMB exhibits a potential anti-atherosclerotic effect by inhibiting macrophage foam cell formation and inflammation. These findings provide novel insights into potential preventive and therapeutic strategies for the clinical management of atherosclerosis.

## Introduction

Cardiovascular diseases (CVD) are the leading causes of mortality globally and contribute significantly to the economic burden of healthcare^1^. Atherosclerosis, a chronic inflammatory disease characterized by the retention of plasma apolipoprotein B (apoB)-containing lipoproteins in focal areas of the arterial tree, serves as the primary pathological basis for CVDs, with risk factors such as hypertension, smoking, hyperglycemia, and hyperlipidemia contributing to its progression^2,3^. Antioxidant, lipid-regulating, and antiplatelet medications are commonly used to treat atherosclerosis. In clinical practice, lipid-lowering agents such as statins, fibrates, ezetimibe, and proprotein convertase subtilisin/kexin type 9 (PSCK9) inhibitors are primarily utilized to prevent and manage atherosclerosis. Although these drugs decelerate disease progression to some extent, their efficacy in reducing cardiovascular mortality is limited to 30%. They are also associated with potential adverse reactions, including respiratory tract infections, muscle pain, low back pain, and joint pain caused by ezetimibe^4^. Given the undesirable effects linked to current atherosclerosis management strategies, there is an urgent need for safer and more efficacious anti-atherosclerotic medications.

Macrophages, vascular smooth muscle cells^5^, endothelial cells^6^, T lymphocytes^7^, fibroblasts^8^, platelets^9^, stem cells^10^, and other cell types are all implicated in the intricate pathogenesis of atherosclerosis. However, macrophages are pivotal in orchestrating this process^11^. In the initial stage of atherogenesis, monocytes are drawn towards the arterial wall by activated-endothelial cells expressing adhesion molecules like intercellular adhesion molecule 1 (ICAM1) and vascular cell adhesion molecule 1 (VCAM1)^12^. Once inside the arterial vessel wall, monocytes differentiate into macrophages exhibiting distinct phenotypes in response to the local immune microenvironment^13^. The pro-inflammatory macrophages efficiently internalize a substantial quantity of LDL-derived cholesterol, thereby facilitating the formation of foam cells^14^. CD36 and scavenger receptor A1 (SR-A1) are the major receptors expressed in macrophages responsible for binding and internalizing modified LDL particles^15^. Cholesterol accumulation in macrophages promotes inflammatory responses, including the activation of Toll-like receptor (TLR) signaling, NF-κB-mediated activation of the NOD-like receptor family, pyrin domain containing 3 (NLRP3) inflammasome, and pro-inflammatory cytokines production, which exacerbate the chronic inflammatory state in atherosclerosis^16^. Increased inflammation attracts more circulating monocytes to the atherosclerotic vessel wall and promotes a vulnerable plaque phenotype^17^. Pro-inflammatory macrophages are known to diminish lesion stability by inhibiting collagen production by smooth muscle cells and producing matrix metalloproteinases (MMPs) that degrade the protective fibrous cap^18^. In summary, the phagocytosis of modified LDL by macrophages and the regulation of inflammation are two pivotal factors implicated in the pathological progression of atherosclerosis.

*Allii Macrostemonis Bulbus*, a renowned traditional Chinese medicine, has been highly valued for its medicinal and dietary properties since ancient times. There are many beneficial compounds in *Allii Macrostemonis Bulbus*, with steroidal saponins being the predominant ones^19^. *Allii Macrostemonis Bulbus* or its prescriptions exhibit various significant activities, including antiplatelet aggregation, lipid-lowering effects, anti-tumor properties, antibacterial activity, and antioxidant potential^20^. Additionally, pharmacological studies have demonstrated that saponins from *Allium macrostemon Bulbus* (SAMB) possess moderate anti-inflammatory effects on endothelial cells through platelet-derived extracellular vesicles, which may be associated with the inhibition of the CD40L/JNK/P38/NF-κB inflammatory signaling pathway^21^. Notably, macrostemon A, a monomer derived from SAMB, has been shown to have therapeutic effects against hyperlipidemia and visceral obesity in mice fed a high-fat diet^22^. However, the effect of SAMB on atherosclerosis and the underlying mechanisms have not been evaluated. This study aimed to determine the impact of SAMB on atherosclerosis in animal models and serve as a proof of concept for the future use of SAMB for the clinical management of atherosclerosis.

## Results

### SAMB inhibited atherosclerosis in ApoE^−/−^ mice induced by a high-fat diet

**Figure 1:**
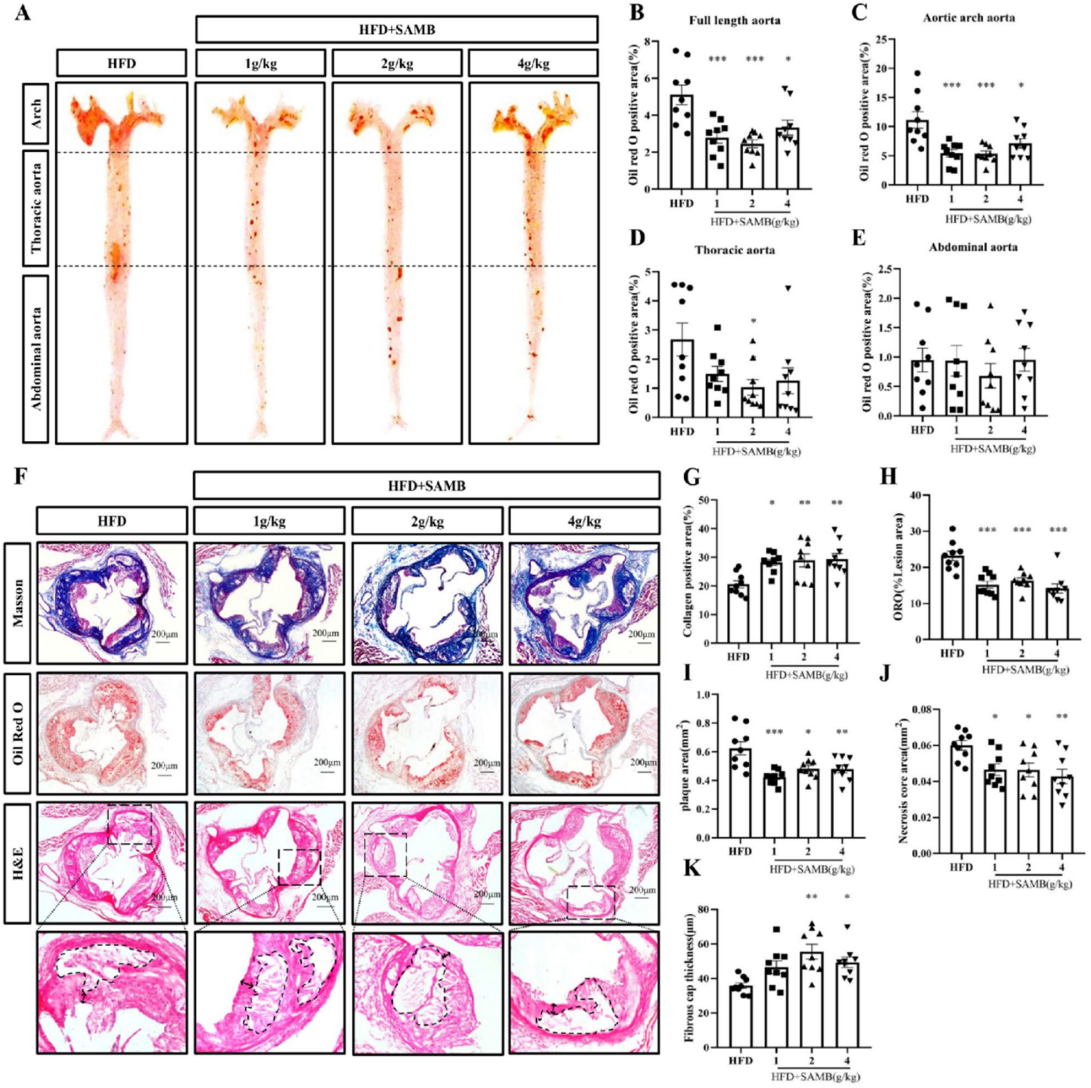
SAMB attenuated atherosclerosis in ApoE^−/−^ mice induced by a high-fat diet. (**A**) Oil-red-O staining of the full-length aorta from the ApoE^−/−^ mice. (**B**–**E**) Quantification of the plaques in the full-length aorta, aortic arch, thoracic aorta, and abdominal aorta. (**F**) Masson, Oil red O, and H&E staining of the aortic root from the ApoE^−/−^ mice (× 200). (**G**,**K**) Quantification of collagen fiber content, intra-plaque fat content, plaque area, necrotic lesion area, and fibrous cap thickness in aortic root plaque. The aortic and root sections of 9 mice in each group were utilized for statistical analysis. Data are mean ± SEM. *P < 0.05, **P < 0.01, ***P < 0.001 vs HFD.

ApoE^−/−^ mice were fed HFD for 14 weeks to investigate the impact of SAMB on atherosclerosis. Compared to the HFD group, all doses of SAMB significantly reduced the lesion area of full-length aortic plaques (Fig. 1A–E). These findings demonstrated that high-, middle-, and low-dose administration of SAMB effectively inhibited HFD-induced lipid deposition in the aorta of HFD-fed ApoE^−/−^ mice. To assess the impact of SAMB on atherosclerotic plaques, various histological staining techniques, including Masson's trichrome, Oil-red-O, and H&E, were employed to evaluate lipid deposition, necrotic core size, fibrous cap thickness, and collagen content. (Fig. 1F–K). Masson's trichrome staining demonstrated a significant increase in collagen fiber content within the aortic root in HFD groups with either high-, middle-, or low-dose SAMB treatment (Fig. 1G), indicating the enhancement of plaque stability by SAMB. Additionally, there was a substantial reduction in lipid deposition within the aortic root plaques among all doses of SAMB treatment assessed by Oil-Red-O staining (Fig. 1H). Furthermore, both plaque area (Fig. 1I) and necrotic area (Fig. 1J) within the aortic root significantly decreased in HFD groups with all three doses of SAMB treatment compared to the model group. Notably, an increased thickness of plaque fibrous cap was explicitly observed in HFD groups with the high- and middle-dose SAMB treatment (Fig. 1K). These findings collectively indicate that administration of SAMB can effectively reduce plaque area within the aortic root and decrease fat content and necrotic foci size within these plaques, thus preventing the initiation and progression of atherosclerotic lesions.

### SAMB reduced serum cholesterol levels in ApoE^−/−^ mice with a high-fat diet feeding

As depicted in Supplementary Fig. S2A, the SAMB group exhibited a slight reduction in body weight compared to the model group. LDL, primarily responsible for cholesterol transportation to peripheral arterial walls during the fasting plasma state, plays a crucial role in promoting atherosclerosis by facilitating the entry and oxidation of small LDL particles^23^. Its concentration is positively associated with coronary heart disease incidence. HDL functions as an anti-atherosclerotic lipoprotein by participating in reverse cholesterol transport and acting as a scavenger within the body. Excessive TC in peripheral tissues can be absorbed by HDL and transported to the liver for excretion, making HDL an essential protective factor against coronary heart disease^24^. Consequently, we assessed TC, TG, LDL-C, and HDL-C serum levels in high-fat-fed ApoE^−/−^ mice (Supplementary Fig. S2B). Compared to the model group, all three doses of SAMB significantly reduced serum TC and TG levels (Supplementary Fig. S2B). The SAMB-high-dose group demonstrated an increased level of serum HDL-C, while both low and middle-dose groups exhibited significant decreases in serum LDL-C concentrations. The findings suggest that SAMB may demonstrate an anti-atherosclerotic effect, partly attributed to its lipid-lowering properties, necessitating further extensive research.

### SAMB suppressed inflammation in ApoE^−/−^ mice fed a high-fat diet

**Figure 2:**
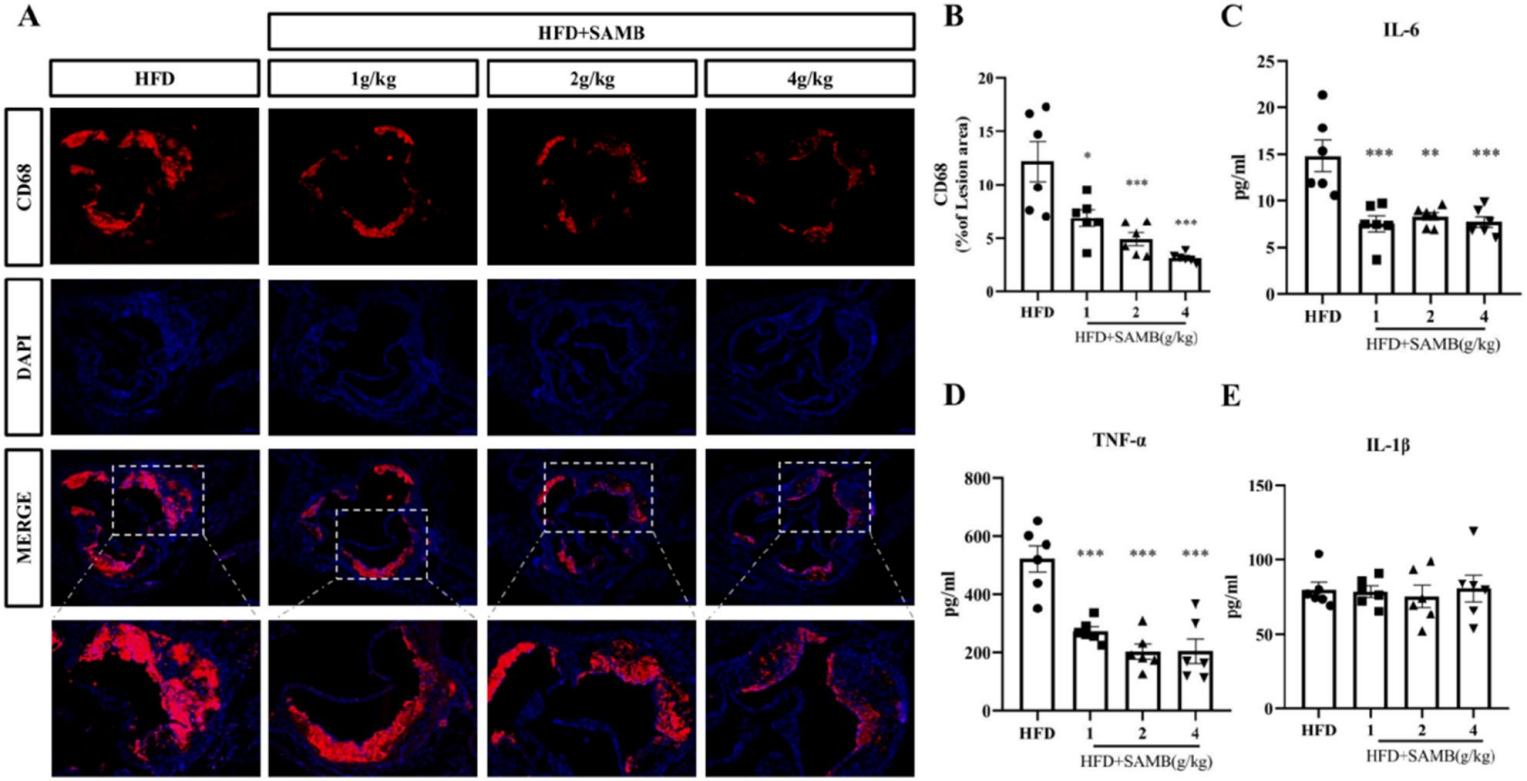
SAMB suppressed the infiltration of macrophages in the aortic root and reduced serum levels of inflammatory factors in the ApoE^−/−^ mice with a high-fat diet feeding. (**A**) CD68 immunofluorescence staining in the aortic root from the ApoE^−/−^ mice (× 200); (**B**) Quantification of the positive staining of CD68 in aortic root plaque. (**C**–**E**) Serum levels of IL-6, TNF-α and IL-1β in the ApoE^−/−^ mice. The aortic root sections of 6 mice in each group were utilized for statistical analysis. Data are mean ± SEM. *P < 0.05, **P < 0.01, ***P < 0.001 vs HFD.

It has been demonstrated that the presence of macrophages within plaques serves as an indicator of atherosclerosis^11^. The infiltration of macrophages into plaques is accompanied by progressive dysfunction, forming plaque necrosis and subsequent shedding^25^. In this study, CD68 immunofluorescence staining was employed to label macrophages in the aortic root (Fig. 2A,B). The results revealed a significant dose-dependent reduction in macrophage infiltration within the plaque area in the aortic root from HFD-fed ApoE^−/−^ mice with SAMB treatment compared to the model group. These findings suggest that SAMB may exhibit an inhibitory effect on macrophage-mediated local inflammation within atherosclerotic plaques in mice.

The presence of inflammatory factors in the bloodstream serves as an indication of a systemic inflammatory response. Inflammatory cytokines play a crucial role in the pathogenesis of atherosclerosis by promoting inflammation and tissue injury, as well as causing endothelial dysfunction and vascular injury, with TNF-α being a key mediator^26^. As depicted in Fig. 2C,D, both high-, middle-, and low-dose SAMB significantly reduced serum levels of IL-6 and TNF-α in HFD-fed ApoE^−/−^ mice. However, no significant effect was observed on serum IL-1β levels between the groups (Fig. 2E). These findings suggest that SAMB may exert an anti-inflammatory effect by reducing the serum levels of inflammatory factors.

### SAMB inhibited the formation of foam cells induced by ox-LDL in bone marrow-derived macrophages (BMDMs)

Macrophage foaming is a prominent hallmark of early atherosclerotic lesions. Excessive uptake of oxidized low-density lipoprotein (ox-LDL) leads to the accumulation of cholesterol esters in macrophages, resulting in the formation of lipid droplets and subsequent foam cell generation^25^. Therefore, in this study, we investigated the impact of SAMB on macrophage foam cell formation in vitro using BMDMs. Before that, a CCK8 assay was conducted to evaluate the cytotoxicity of SAMB, revealing that concentrations below 1mg/ml exhibited negligible cytotoxic effects on BDMDs within a 48-h timeframe (Supplementary Fig. S3).

**Figure 3:**
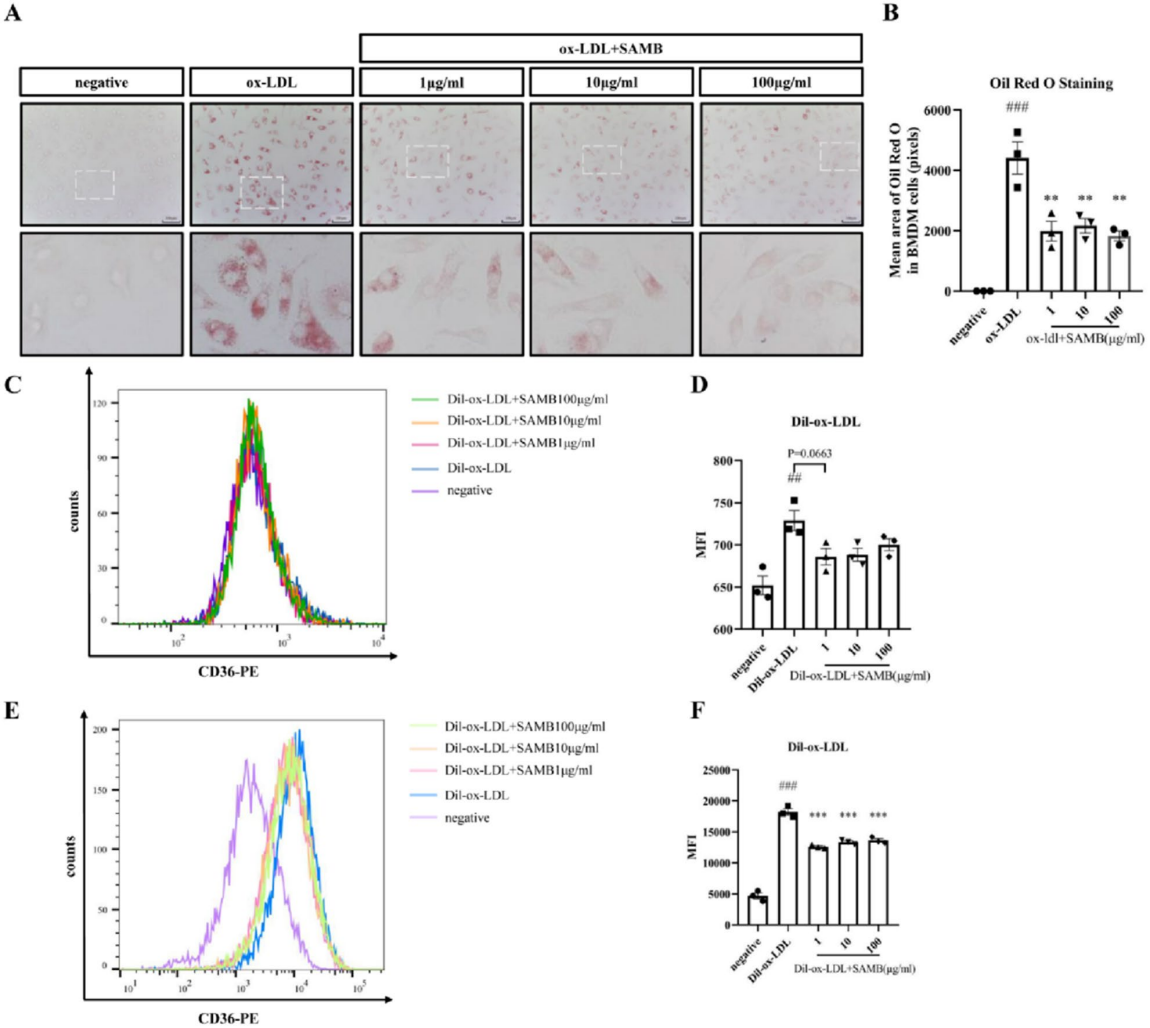
SAMB inhibited the foam cell formation by down-regulating the ox-LDL endocytosis in BMDMs. (**A**) Oil-red-O staining of BMDMs (× 400). (**B**) Quantification of the positive Oil-red-O staining in BMDMs. (**C**) Flow cytometry of DiI-ox-LDL binding to BMDMs. (**D**) Quantification of BMDMs with bound DiI-ox-LDL. (**E**) Flow cytometry of Dil-ox-LDL internalization by BMDMs. (**F**) Quantification of Dil-ox-LDL internalization (or uptake) by BMDMs. The quantification of Dil-ox-LDL was performed using MFI (mean fluorescence intensity). Data are mean ± SEM. ^##^P < 0.01 and ^###^P < 0.001 vs negative control; **P < 0.01 and ***P < 0.001 vs ox-LDL.

Compared to the negative control group, ox-LDL incubation caused a significant lipid deposition in BMDMs assessed by Oil-Red-O staining, which was effectively suppressed by high, medium, or low doses of SAMB (Fig. 3A,B). This result demonstrates that SAMB at different doses can effectively inhibit macrophage foam cell formation. However, whether this effect is achieved through the modulation of macrophage adhesion to or endocytosis of ox-LDL remains uncertain.

BMDMs were incubated with the Dil-ox-LDL at 4 °C for 1 h, followed by flow cytometry analysis to investigate the impact of SAMB on ox-LDL binding to the macrophage membrane surface. Figure 3C,D show that the mean fluorescence intensity (MFI) of macrophages in the Dil-ox-LDL group was significantly higher than in the negative control group. At the same time, only a downward trend was observed in the Dil-ox-LDL + SAMB group, which had no statistical significance compared to the Dil-ox-LDL group. These findings indicate that SAMB may not affect the binding of Dil-ox-LDL to BMDMs surface.

To investigate the impact of SAMB on lipoproteins internalization in BMDMs, BMDMs were incubated with Dil-ox-LDL at 37 °C for 8 h and then washed with acidic PBS to remove the Dil-ox-LDL bound to the cell membrane surface, followed by flow cytometry analysis. Compared to the negative group, a significant increase in macrophage MFI was observed in the Dil-ox-LDL group, in which SAMB markedly reduced in either high, middle, or low doses (Fig. 3E,F). These findings suggest that SAMB effectively inhibited ox-LDL internalization in macrophages.

### SAMB suppressed the expression of cholesterol transport-related genes in BMDMs

**Figure 4:**
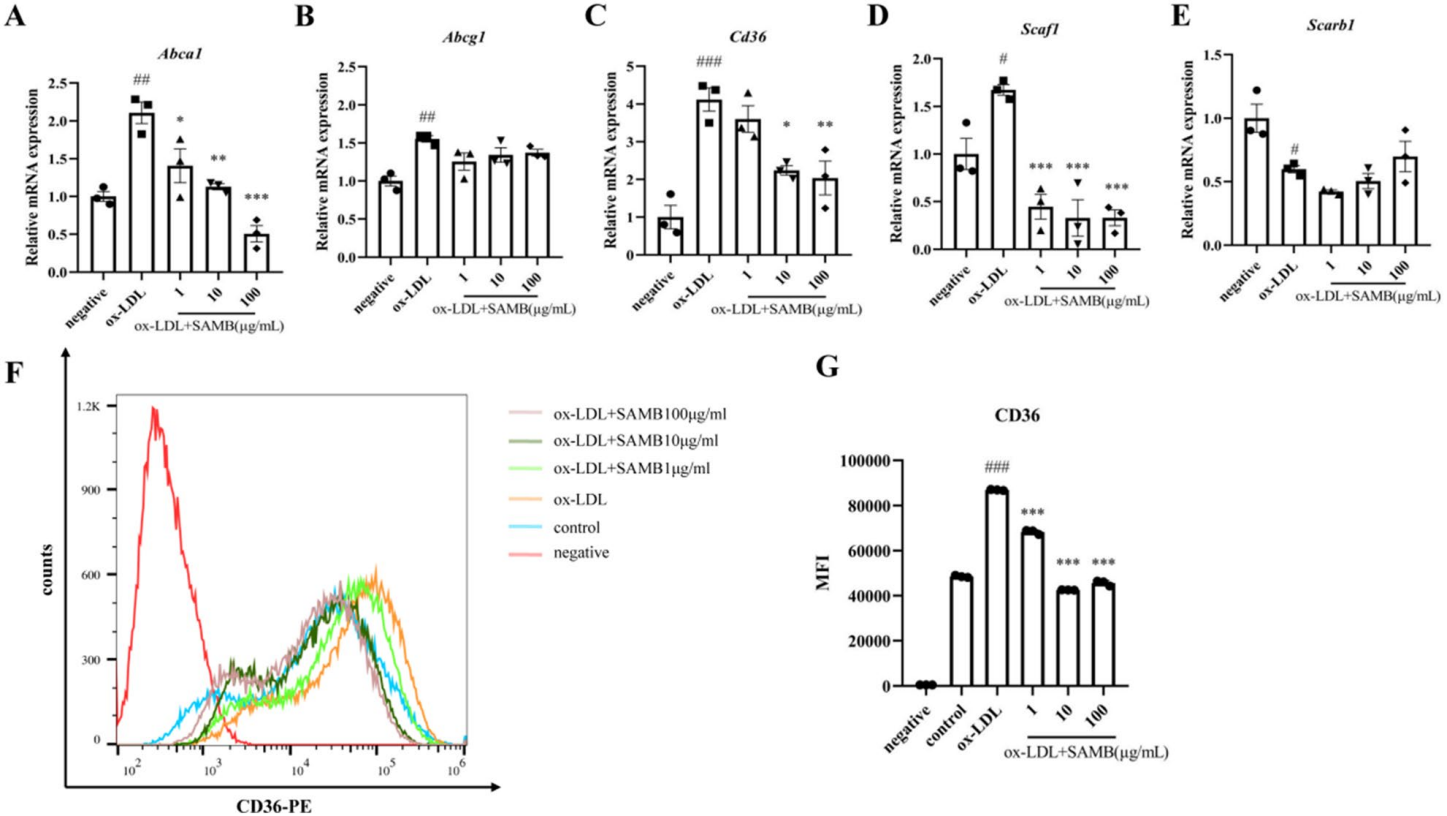
SAMB regulated the lipoprotein transporter mRNA expression and significantly suppressed the CD36 expression in ox-LDL-incubated BMDMs. (**A**–**E**) mRNA expression levels of *Abca1*, *Abcg1*, *Cd36*, *Scaf1* and *Scarb1* in BMDMs assessed by RT-qPCR. (**F**) Streaming graph of CD36 expression on BMDMs surface assessed by flow cytometry. (**G**) Quantification of CD36 expression on BMDMs surface. Three independent experiments were conducted. Data are mean ± SEM. ^#^P < 0.05, ^##^P < 0.01, ^###^P < 0.001, and ^####^P < 0.0001 vs negative control; *P < 0.05, **P < 0.01, ***P < 0.001 vs ox-LDL.

To elucidate the mechanism underlying SAMB's impact on macrophage foam cell formation, the expression of cholesterol transport-related genes was determined by RT-qPCR. CD36 and SR-A1 facilitate cholesterol uptake, while ABCA1, ABCG1, and SR-B1 are associated with the excretion of cholesterolcholesterol excretion from macrophages to the extracellular environment^27^. To investigate the impact of SAMB on lipid transport in macrophages, we examined the expression levels of cholesterol transport-related genes using qPCR. As shown in Fig. 4A–E, ox-LDL significantly upregulated the mRNA expression levels of *Abca1*, *Abcg1*, *Cd36*, and *Scaf1* but downregulated *Scarb1* mRNA levels compared to the negative control group. Furthermore, both high-, middle-, and low-dose SAMB significantly decreased the mRNA expression levels of *Scaf1* and *Cd36* compared to the ox-LDL group. Moreover, SAMB also downregulated *Abca1* mRNA expression without significantly affecting *Abcg1* or *Scarb1* mRNA expression. These findings suggest that SAMB may inhibit atherosclerosis by attenuating lipid influx into macrophages from extracellular sources.

Scavenger receptor CD36, a single-stranded transmembrane glycoprotein belonging to the class B receptor family, primarily facilitates the recognition and uptake of ox-LDL^28^. To elucidate the mechanism underlying SAMB's impact on macrophage foam cell formation, we employed flow cytometry to assess the levels of CD36 on the surface of BMDMs. Compared to the negative control group, a significant increase in macrophage MFI was observed in the ox-LDL group, which was significantly attenuated by SAMB (Fig. 4F,G). These results indicated the inhibitory effect of SAMB on CD36 expression on macrophage surfaces.

### SAMB inhibited LPS-induced inflammation by inhibiting the NF-κB/NLRP3 pathway in BMDMs

**Figure 5:**
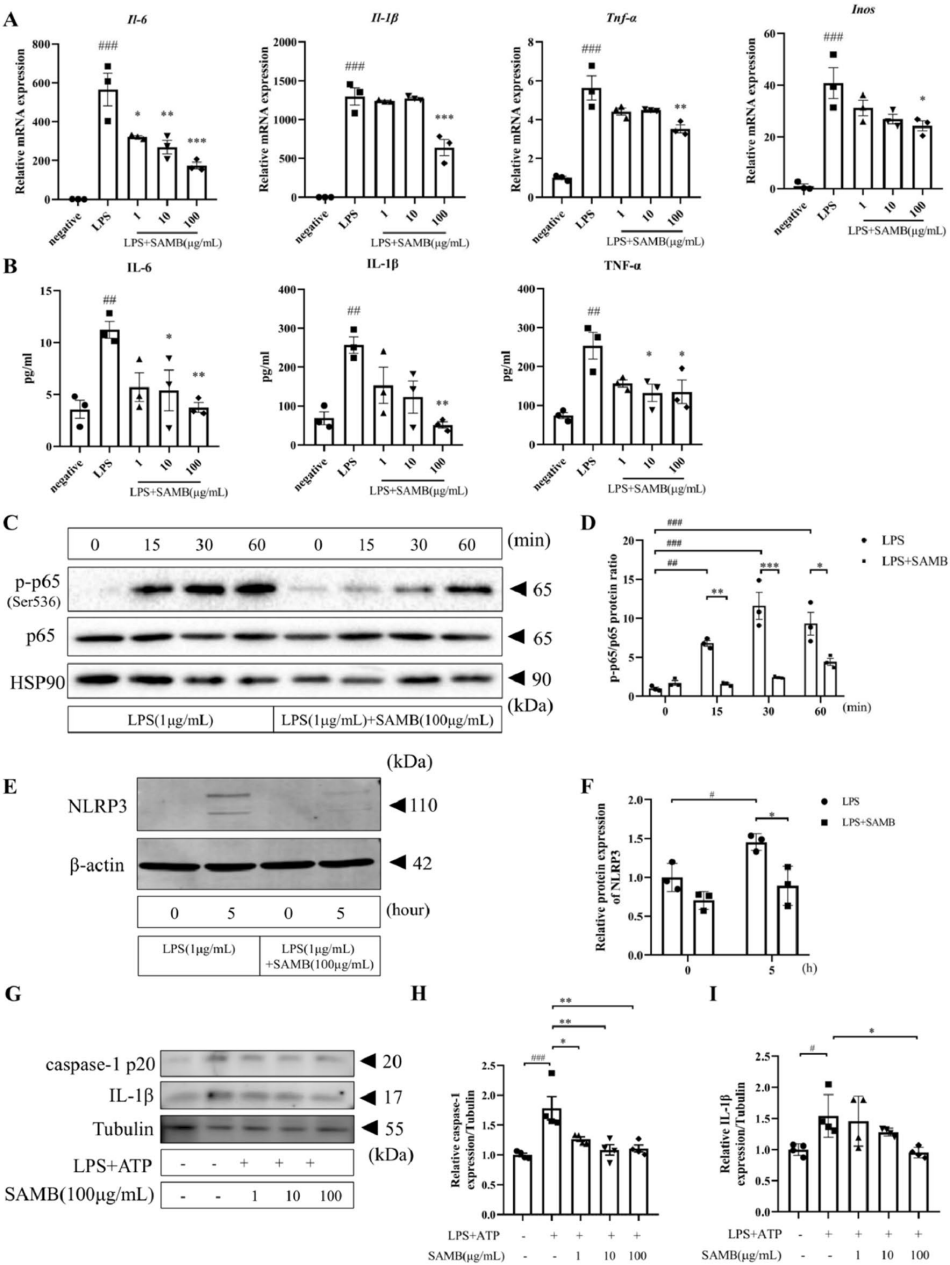
SAMB inhibited LPS-induced inflammation through inhibiting NF-κB/NLRP3 pathway in BMDMs. (**A**) *Il-1β*, *Il-6*, *Tnf-α*, and *Inos* mRNA levels assessed by RT-qPCR. (**B**) IL-1β, IL-6, and TNF-α levels in cell culture medlum of BMDMs assessed by ELISA. (**C**,**D**) The effect of SAMB on NF-κB p65 and p-NF-κB p65 protein expression. (**E**,**F**) The effect of SAMB on NLRP3 protein expression. (**G**–**I**) The effect of SAMB on caspase-1 p20 and IL-1β expression in the inflammasome model. Data are mean ± SEM. ^#^P < 0.05, ^##^P < 0.01, ^###^P < 0.001 vs negative; *P < 0.05, **P < 0.01, ***P < 0.001 vs LPS.

Upon LPS stimulation, macrophages exhibit the expression of an array of inflammatory factors. After binding to the plasma's LPS-binding protein (LBP), LPS is transported to the cell surface receptor CD14. Then, it is transferred to receptors along with its accessory protein MD2. It will activate multiple signaling pathways, including IκB kinase (IKK)-NF-κB pathway and mitogen-activated protein kinases (MAPK) pathways^29^. These pathways activate various transcription factors such as NF-κB (P50/P65), c-Jun, and STAT1, leading to the upregulation of inflammatory factors like TNF-α, IL-1β, IL-6, iNOS, and NLRP3^30^. SAMB at high-, medium-, and low doses significantly suppressed LPS-stimulated *Il-6* mRNA expression in BMDMs. However, the high-dose but not the medium- or low-dose SAMB markedly reduced *Tnf-α, Il-1β*, and *Inos* mRNA levels in LPS-treated BMDMs (Fig. 5A). The TNF-α, IL-1β, and IL-6 levels in the cell culture medium were quantified using ELISA, yielding similar results (Fig. 5B). Additionally, SAMB significantly decreased phosphorylated NF-κB p65 (Fig. 5C,D) and NLRP3 (Fig. 5E,F) protein expression in LPS-stimulated BMDMs assessed by western blotting. However, no significant effects were observed on the extracellular regulated protein kinases (ERK) and c-Jun N-terminal kinase (JNK) pathway (Supplementary Fig. S4). To further elucidate the inhibitory effect of SAMB on NLRP3-mediated inflammasome activation, we established an inflammasome model by sequentially stimulating BMDMs with LPS and ATP, followed by quantification of intracellular IL-1β and caspase-1 p20 levels using Western blotting analysis. The results demonstrated a significant increase of both IL-1β and caspase-1 p20 in the model group, which was significantly suppressed by SAMB (Fig. 5G***), suggesting the inhibitory action of SAMB on inflammasome activation.

Collectively, our findings suggest that SAMB may possess potential therapeutic efficacy against atherosclerosis by exerting anti-inflammatory effects via the modulation of the NF-κB/NLRP3 signaling pathway.

## Discussion

**Figure 6:**
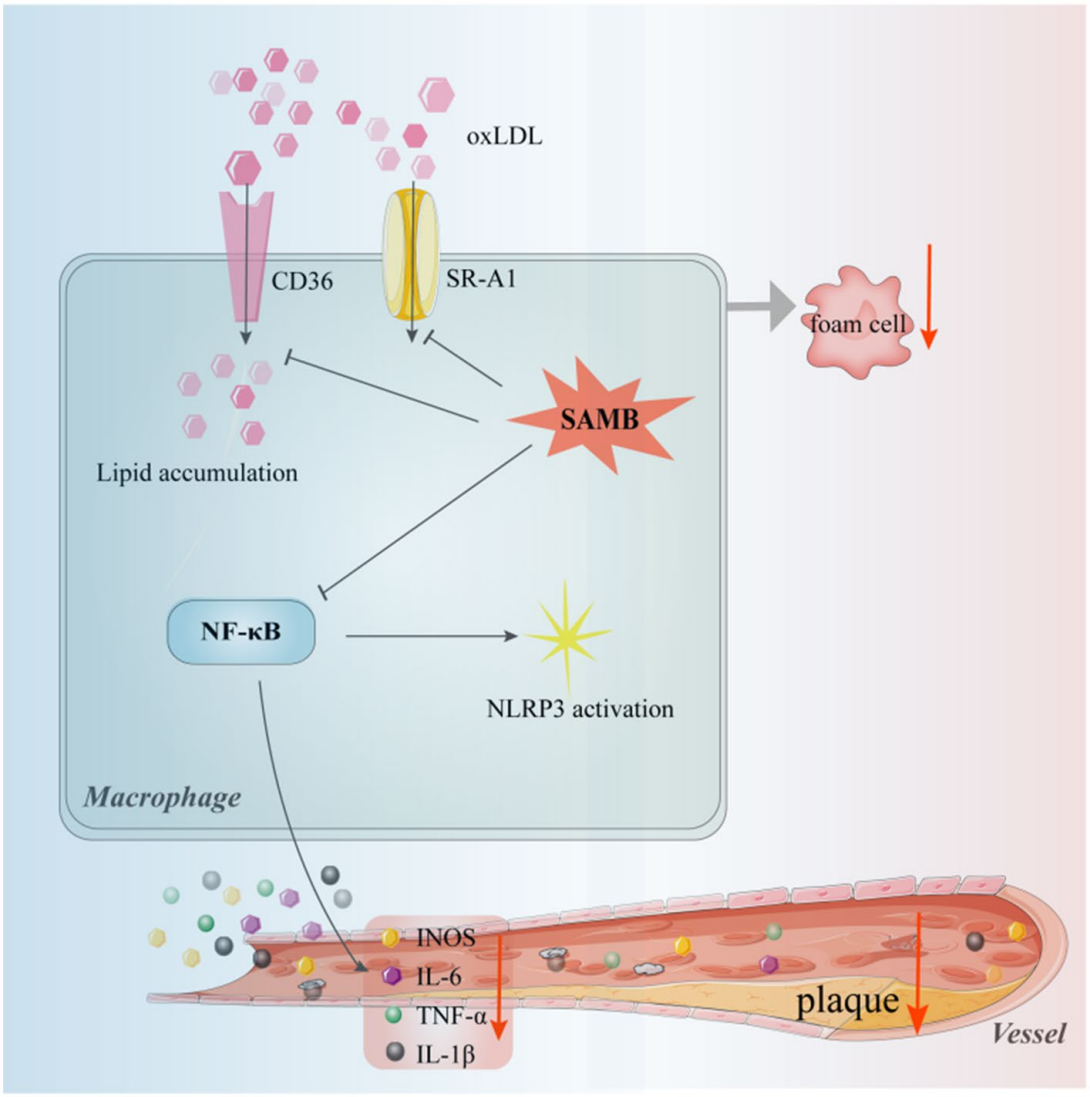
A proposed model for the role of SAMB in regulating atherosclerosis. On one hand, SAMB can downregulate the translation and transcription of lipid transporters CD36 and SR-A1, thereby inhibiting the phagocytosis of oxidized low-density lipoprotein by macrophages, reducing foam cell formation and plaque development. On the other hand, SAMB can suppress the expression of inflammatory factors by inhibiting the NF-κB/NLRP3 signaling pathway, resulting in decreased macrophage infiltration in plaques.

Our current study aimed to investigate SAMB's potential anti-atherosclerotic effects in an HFD-induced atherosclerosis model. Our data have demonstrated that the application of SAMB protected against atherosclerosis, as reflected by the significant reduction of plaque area within the aorta and the serum cholesterol levels, as well as the inhibition of foam cell formation, cholesterol influx, and inflammation in the macrophages. Mechanistically, the protective action of SAMB may be associated with inhibiting the NF-κB/NLRP3 pathway in the macrophages (Fig. 6). Our findings provide valuable insights for the potential application of SAMB in patients with atherosclerosis, highlighting the need for clinical assessment of its efficacy in protecting against plaque formation in individuals with atherosclerotic conditions.

Atherosclerotic cardiovascular disease is a prevalent cause of morbidity and mortality worldwide, necessitating effective interventions to reduce the risk of cardiovascular events^1^. Despite treatment with lipid-lowering therapies, such as statins and PCSK9 inhibitors, the incidence of these events remains high in the general population^31^. Theoretically, early reduction of apoB-containing lipoproteins to very low levels could potentially eliminate cardiovascular disease; however, this strategy currently faces practical limitations due to issues like low compliance and adverse effects in certain individuals^32^. The *Allii Macrostemonis Bulbus*, first documented in the renowned Chinese herbal classic Shen-Nong-Ben-Cao-Jing, is widely recognized as a superior herb for treating thoracic obstruction and cardiodynia (resembling coronary heart disease)^33^. Additionally, *Allii Macrostemonis Bulbus* exhibits a high level of bioactivity safety, as it is recognized as a permissible food item under the Food Safety Law of the People's Republic of China due to its exceptional nutritional value^34^. The chemical components of *Allii Macrostemonis Bulbus* are typically classified as volatile oils, nitrogenous compounds, steroidal saponins, and other constituents. SAMB exhibits remarkable biological properties among these compounds and holds great potential for treating atherosclerosis^35^. Along this line, in the present study, we first reported that oral administration of SAMB significantly ameliorated HFD-induced atherosclerosis in ApoE^−/−^ mice.

The initiation, progression, and regression of atherosclerotic lesions are influenced by macrophages^36^. Macrophages within atherosclerotic plaques exhibit a foamy phenotype and phenotypic heterogeneity (M1 or M2) due to the abundance of oxidized lipids and lipoproteins in the plaque microenvironment^37^. Consequently, macrophage dysfunction plays a pivotal role in the advancement of atherosclerosis. Our findings in the present study demonstrated that treatment with SAMB led to reduced uptake of ox-LDL by BMDMs and decreased formation of foam cells, indicating the inhibitory action of SAMB on foam cell formation by attenuating lipid internalization in macrophages. The formation of foam cells is also regulated by genes involved in cholesterol transport. Notably, CD36 (a highly specific receptor for ox-LDL) and SR-A1 are crucial in facilitating cholesterol influx into macrophages^38^. Upon activation by ox-LDL, CD36 triggers signaling cascades and upregulates its expression, thereby promoting ox-LDL uptake^39^. Disruption of the balance between cholesterol influx and efflux eventually transforms macrophage into foam cells. We found that SAMB significantly inhibited the transcriptional levels of CD36 and SR-A1 in ox-LDL-incubated BMDMs. Interestingly, SAMB also downregulated the mRNA expression of *Abca1*, an essential cholesterol efflux transporter. We speculate that this is a consequence of the attenuated CD36-mediated lipid influx and overall intracellular lipid content in the SAMB-treated macrophages. Furthermore, the inhibitory effect of SAMB on CD36 expression was confirmed at both mRNA and protein levels, providing preliminary insights into the potential molecular mechanism for the anti-atherosclerotic action of SAMB through the regulation of lipid metabolism-related targets in the macrophages. The specific mechanisms of SAMB regulating CD36 await future investigations.

The accumulation of foam cells produces pro-inflammatory factors such as IL-1β, IL-6, TNF-α, chemokines, and growth factors. Various inflammation-related signals are activated upon stimulation by these pro-inflammatory factors, with the NF-κB signaling pathway playing a crucial role^40^. Overactivation of the NF-κB pathway has been observed in atherosclerosis patients and animal models, implying an effective treatment strategy for atherosclerosis by inhibiting the NF-κB pathway^41^. Here, we also investigated the effect of SAMB on the NF-κB activity in LPS-stimulated BMDMs by examining NF-κB p65 phosphorylation. SAMB significantly reduced phosphorylated NF-κB p65 and the levels of pro-inflammatory factors, including IL-6, TNF-α, and IL-1β in LPS-treated BMDMs. Indeed, we previously utilized the NF-κB inhibitor BAY-117082 to investigate whether SAMB achieved anti-inflammatory effects by inhibiting NF-κB signaling in human umbilical vein endothelial cells (HUVECs)^42^. Our findings suggested that SAMB's downregulation of VCAM-1 expression in endothelial cells, a key inflammatory factor, may be attributed to the inhibition of the NF-κB pathway^42^. These results also allow us to speculate that SAMB may exert a similar anti-inflammatory action by inhibiting the NF-κB pathway in BMDMs. Thus, these results provide preliminary insights into SAMB's anti-inflammatory effect, which may explain its anti-atherosclerotic action. Of note, endothelial dysfunction is recognized as an underlying cause of atherosclerosis. The anti-atherosclerotic action of SAMB may also be attributed to its inhibitory role on endothelial dysfunction, which was possibly supported by the suppressive impact of SAMB on VCAM-1 expression in endothelial cells^42^. Thus, further studies detecting endothelial dysfunction markers such as ZO-1, ICAM-1, and VCAM-1 in the aortic roots by immunofluorescence staining are recommended.

As a class B scavenger receptor family member, CD36 can recognize various endogenously derived hazardous molecules (e.g., ox-LDL), thereby triggering the initiation of sterile inflammation^43^. CD36 is implicated in priming and activating NLRP3 inflammasome^42,43^. Sheedy et al. demonstrated that macrophage CD36 sequestered ox-LDL, leading to the activation of NF-κB downstream of the heterotrimeric CD36-TLR4-TLR6 complex, thereby inducing up-regulation of NF-κB-driven NLRP3 expression^44^. Furthermore, ox-LDL uptake through CD36 leads to the accumulation of cholesterol crystals within cells, resulting in lysosomal disruption and subsequent activation of NLRP3-inflammasome^45^. Along with this line, inhibition of CD36 effectively suppresses the activation of NLRP3 inflammasome^28^. Thus, CD36 in macrophages may be a pivotal molecule associated with foam cell formation and the development of chronic sterile inflammation in atherosclerosis. In support of this notion, the present study convincingly demonstrated a significant downregulation of p-NF-κB p65 and NLRP3 protein expression by SAMB treatment in LPS-stimulated BMDMs, indicating the inhibitory effect of SAMB on inflammasome formation. In LPS/ATP-induced inflammasome model, SAMB treatment resulted in a significant decrease of both IL-1β and caspase-1 p20, providing compelling evidence for the inhibitory effect of SAMB on inflammasome activation. Future studies using confocal microscopy to verify the anti-inflammatory effects of SAMB on NLRP3, ASC, or Caspase 1 expression are warranted.

Some critical issues remain to be further clarified. First, the oral bioavailability of SAMB is unclear. Second, the specific active ingredients of SAMB are unknown. Lastly, the precise target of SAMB's anti-atherosclerotic action is still unknown; specifically, the CD36-dependency mechanisms for the effects of SAMB need to be elucidated.

In conclusion, SAMB ameliorates atherosclerosis by inhibiting foam cell formation and inflammation. These findings provide novel insights into potential preventive and therapeutic strategies for clinical management of atherosclerosis.

## Materials and methods

### Materials and chemicals

The *Allii Macrostemonis Bulbus* were obtained from Jiangxi Jiangzhong Prepared Slices of Chinese Crude Drugs Co., Ltd. Dimethyl sulfoxide (D8370), Trypsin (T8150), Oil red O powder (O8020), high sugar Dulbecco's modified Eagle's medium (DMEM) (12,100–500), and PMI 1640 medium (31,800) were purchased from Solarbio (Beijing, China). CCK8 (BS350B) was obtained from Biosharp (Hefei, China). FBS (CC-4101A) was acquired from Lonza (Walkersville, MD, USA). Human ox-LDL (yb-002) and fluorescent-labeled ox-LDL (yb-0010) were obtained from Yiyuan Biology (Guangzhou, China). CD68 antibody (MCA1957), CD36 antibody (562,702), and NLRP3 antibody (AG-20B-0014-C100) were respectively purchased from Bio-Rad (Kidlington, USA), BD Biosciences (San Jose, CA, USA), AdipoGen (Berne, CH). p-p65 antibody (3033S) and p65 antibody (6956S) were purchased from CST (Danvers, MA, USA). Blood lipid test kits were procured from Nanjing Jiancheng Bioengineering Institute.

### Animals

The animal studies were approved by the Institutional Animal Care and Use Committee of the Jiangxi University of Chinese Medicine (No. JZLLSC20230254), and all the processes are in strict accordance with the National Institutes of Health (NIH) Guide for the Care and Use of Animals in laboratory experiments. All methods were reported following ARRIVE guidelines. The ApoE^−/−^ mice (SPF grade) were purchased from Nanjing Biomedical Research Institute of Nanjing University [Certificate of Conformity No. SCXK(Su) 2015-0001]. A total of 36 male mice, aged 6–8 weeks and weighing approximately 20 g each, were utilized for the study. These mice were bred in a controlled environment at the Laboratory Animal Science and Technology Center of Jiangxi University of Chinese Medicine under specific pathogen-free conditions before being transferred to individual ventilated cages (IVC). Mice were housed in a temperature (20–26 °C)- and humidity (40–70%)-controlled room with a 12:12 h light–dark cycle.

Male ApoE^−/−^ mice aged 6–8 weeks were fed a high-fat diet (HFD) and randomly assigned to four groups (n = 8 or 9): HFD group treated with 100 µL sterile PBS (0.01 M); HFD group treated with low (1 g/kg of body weight), medium (2 g/kg of body weight), and high doses (4 g/kg of body weight) of SAMB solution, respectively. All mice were orally administered either PBS or SAMB once daily for 14 weeks via gavage. The mice were anesthetized by intraperitoneal injection of ketamine (100 mg/kg) and xylazine (10 mg/kg) before sacrifice. Serum, heart, full-length aorta, and liver samples were harvested from the mice for further analysis.

### Preparation of SAMB extract

The dry *Allium macrostemon* (40 g) was weighed, crushed, and sifted through a 10-mesh sieve. It was soaked for half an hour and extracted three times with 8 times, 6 times, and 4 times the volume of 60% ethanol at an extraction temperature of 60–70 °C for each period of 0.5 h. The resulting ethanol extract was centrifuged at 2600 rpm/min for 20 min to discard the residue and sediment. The supernatant was combined and concentrated using rotary evaporation until reaching a final volume within the 20–30 mL range. Subsequently, the *Allium macrostemon* extract was adsorbed onto an HP-30 macroporous resin column for three hours. Subsequently, it was eluted using a five-fold volume of 30% ethanol, followed by 60% ethanol, and finally 95% ethanol. The eluents were collected, concentrated, and subjected to identification via TLC analysis (Supplementary Fig. S1). Specifically, the extract obtained from the elution with 60% ethanol was designated the SAMB solution. The SAMB content was quantified using diosgenin as the reference standard, revealing that approximately 3.93 mg of SAMB could be extracted from every 1 g of *Allium macrostemon*. Additionally, the constituents of SAMB were analyzed utilizing iEESI-MS (Internal Extractive Electrospray Ionization Mass Spectrometry), leading to the identification of a total of 15 saponins (Supplementary Table S1).

### Detection of full-length aortic plaque area by Oil-Red-O staining

The fat-stripped full-length aorta was fixed overnight in a 4% PFA solution. Subsequently, the vessel underwent two equilibration steps of 5 min each with 60% isopropanol. Staining with 0.3% Oil red O staining solution was conducted at room temperature for 20 min under dark conditions. Differentiation occurred briefly in 60% isopropanol before terminating the staining process. Excess dye was removed by rinsing with distilled water, and longitudinally cut blood vessels were spread onto glass slides for microscopic examination and documentation.

### Histopathological staining and immunofluorescence analysis

The aortic roots were fixed in 4% PFA and washed with PBS. Subsequently, the samples were embedded in an optimal cutting temperature compound and frozen using liquid nitrogen. Cryosections measuring 10 µm in thickness were obtained from the aortic root to the apex. A set of sections was collected on a stereomicroscope slide and stained with Oil red O, hematoxylin–eosin, and Masson's trichrome (Solarbio, China). The sections stained with Oil red O received counterstaining with hematoxylin–eosin for 30 s. After a 2-min tap water wash, glycerin and gelatin were used to mount the sections before capturing images using Nikon's digital camera model 4,500. Image J software (V1.8.0, https://imagej.en.softonic.com) was utilized to determine the sizes of both atherosclerotic plaque and collagen fibers.

The frozen sections were rinsed with PBS solution for 15 min, followed by blocking in a solution containing 5% donkey serum, 0.5% bovine serum albumin (BSA), and 0.03% Triton X-100 for 1 h at room temperature. Subsequently, the sections were incubated overnight at 4 °C with primary anti-CD68 antibody (1:250 dilution) and secondary antibody (1:500 dilution). After another round of rinsing with PBS solution for 15 min, the sections were incubated with fluorescence-conjugated secondary antibody for an additional hour and stained with DAPI for three minutes. Following the removal of the DAPI solution, the sections were imaged using fluorescence microscopy, and the average fluorescence intensity was quantified using Image J software by calculating the total area of fluorescence intensity relative to the plaque area.

### Isolation of mouse bone marrow-derived macrophages

As previously described, bone marrow cells were isolated from femurs and tibias of 8-week-old C57BL/6 mice^46^. L929 cells were cultured in RPMI 1640 medium and incubated in a humidified atmosphere (5% CO_2_; 37 °C) for 5 days. The culture media was then collected and centrifuged at 1000 rpm for 5 min, and the supernatant was harvested as the L929 conditioned medium. Eight-week-old male C57BL/6 mice were sacrificed and immersed in a sterilizing solution of 75% ethanol for 5 min. Bilateral femurs were separated and washed with a macrophage starvation medium. Then, the ends of the femurs were removed, and bone marrow from the femurs was flushed out using DMEM. Cells were centrifuged at 3000 rpm for 5 min and suspended in DMEM supplemented with 10% fetal bovine serum, penicillin (100 U/mL), and streptomycin (100 U/mL). Subsequently, cells were plated onto 10-cm dishes and incubated in a humidified atmosphere (5% CO_2_; 37 °C) for 72 h. Floating cells in the medium were collected and centrifuged. The harvested cells were resuspended with L929 conditioned medium and adhered to the cell dish, and then the medium was replaced.

### Cell viability assay

BMDMs were cultured in 96-well plates to reach sub-confluence, and then they were treated with different concentrations of SAMB for 24, 48, and 72 h, respectively. Subsequently, 10 µL of CCK-8 was added into each well to incubate cells for another 1 h at 37 °C with 5% CO_2_. Absorbances at 450 nm were measured using a microplate reader (Bio-Rad).

### Foam cell formation assay

The BMDMs obtained as described above were inoculated in 24-well plates and arranged as follows: the blank group (vehicle), the lipoprotein group (ox-LDL), and the lipoprotein + SAMB low-, medium-, and high-dose groups (ox-LDL + 1, 10, and 100 μg/mL SAMB). The final concentration of ox-LDL was set at 80 μg/mL. After a 24-h incubation period, the medium in each well was aspirated, and the wells were washed twice with PBS. Subsequently, cells were fixed with a solution of 4% PFA at room temperature for 30 min. PFA was then removed by rinsing three times for five minutes each time. Following a brief equilibration step with 60% isopropanol for five minutes, Oil red O staining (1 mL/well) was performed in the dark at room temperature for 25 min. The oil red O solution was discarded, and excess dye was removed using 60% isopropanol, followed by three washes lasting three seconds each time. The cell counting under a microscope was finally performed, with the number of cells in each image ranging from 60 to 90.

### Analysis of Dil-oxLDL binding and uptake

Binding assay: BMDMs were seeded in 12-well plates and incubated with SAMB and Dil-oxLDL at 4 °C for 1 h to analyze the binding.

Uptake assay: BMDMs were stimulated with SAMB and Dil-oxLDL for 8 h at room temperature to assess the uptake process. Following stimulation, cells were washed four times with acidic PBS for 5-min intervals. Subsequently, trypsin digestion was performed for 5 min, followed by centrifugation at 1000 rpm for three minutes. The resulting cell pellets were resuspended in PBS before being transferred to sample tubes for flow cytometry analysis.

The binding assay and uptake assay were each conducted in triplicate, yielding a total of three independent experiments.

### Quantitative RT-PCR

Total RNA was extracted from cells or tissues using Trizol reagents, followed by cDNA synthesis using the PrimeScript RT reagent Kit (Takara, Kyoto, Japan) according to the manufacturer's instructions. Real-time PCR was performed with SYBR R Premix Ex Taq II (Takara, Kyoto, Japan), and primer sequences are provided in Supplementary Table S2. β-actin served as an internal control.

### Flow cytometric assay

SAMB was added to macrophages for flow cytometric assay, followed by administration of oxidized low-density lipoprotein (ox-LDL) at a concentration of 80 μg/mL after 24 h. Immediately after incubating, the medium was removed, and the cells were washed four times with PBS. The cells were then scraped off using a spatula and suspended in 1 mL PBS. After centrifugation at 1200 rpm for 5 min, the supernatant was discarded, and suspensions in 100 μL of PBS containing 2% FBS were transferred to a flow tube. A dilution of CD36 at a ratio of 1:200 was incubated in the dark at 4 °C for 30 min. Subsequently, centrifugation at 1200 rpm for another 5 min was performed, followed by resuspension of pellets with the addition of 400 μL PBS. The current and voltage settings on the flow cytometer were adjusted upon turning it on using the blank group as a reference. The enclosed cells without fluorescence expression were set as negative cell population gates, while outside cells with fluorescence labeling constituted positive cell population gates; counting positive cells and mean fluorescence intensity measurement took place after loading completion.

### Inflammasome model

The inflammasome model was established by stimulating BMDMs with a combination of LPS and ATP. Briefly, BMDMs were stimulated by 2 μg/mL LPS for 5 h following a 24-h incubation period with SAMB, followed by subsequent stimulation with 5 mM ATP for 45 min. The cells were subsequently harvested for western blotting analysis to evaluate the caspase-1 p20 and IL-1β intracellular levels.

### Western blotting

Cells were homogenized using 150 µL of RIPA lysis buffer, followed by centrifugation (12,000 rpm, 15 min, 4 °C). Protein quantification was performed in the supernatant using a BCA protein assay kit. The precise amount of 40 µg of total protein was separated via SDS-PAGE and transferred onto polyvinylidene fluoride membranes with a pore size of 0.45 µm for subsequent overnight probing at 4 °C. Subsequently, the membranes were incubated with secondary horseradish peroxidase-conjugated antibodies (anti-mouse or anti-rabbit) for one hour at room temperature. Immunoblots were visualized using Image Studio.

### Statistical analysis

All values were expressed as the mean ± S.E.M and analyzed using GraphPad Prism 10 (https://www.graphpad.com). One-way analysis of variance (ANOVA) followed by Dunnett's test was used to evaluate statistical differences among groups. A P-value < 0.05 is considered statistically significant.

### Supplementary Information

Supplementary Information.

## Data Availability

The raw data supporting the conclusions of this article will be made available by Prof. Yanfei Xie or Prof. Chuanming Xu without undue reservation.
