# Supplementary material for: Saponins from Allii Macrostemonis Bulbus attenuate atherosclerosis by inhibiting macrophage foam cell formation and inflammation
Source: Sci Rep. 2024 Jun 5;14:12917. doi: 10.1038/s41598-024-61209-w (PMC11153636; doi:10.1038/s41598-024-61209-w)

**Saponins from *Allii Macrostemonis Bulbs* Attenuates Atherosclerosis by Inhibiting Macrophage Foam Cell Formation and Inflammation**

Shutian Zhao^1^, Huijun Guo^1^, Liang Qiu^1^, Chao Zhong^1, 2^, Jing Xue^1^, Manman Qin^1^, Yifeng Zhang^1^, Chuanming Xu^1,^ *, Yanfei Xie^1,^ *, Jun Yu^2^

^1^Translational Medicine Centre, Jiangxi University of Chinese Medicine, Nanchang, Jiangxi, China

^2^Department of Cardiovascular Sciences and Center for Metabolic Disease Research, Lewis Katz School of Medicine, Temple University, Philadelphia, PA, USA

**Running title:** SAMB protects against atherosclerosis

*Correspondence:

Yanfei Xie, PhD

Translational Medicine Centre, Jiangxi University of Chinese Medicine, Nanchang, Jiangxi, China

E-mail: 1047501830@qq.com

Or

Chuanming Xu, PhD

Translational Medicine Centre, Jiangxi University of Chinese Medicine, Nanchang, Jiangxi, China

E-mail: xuchuanming2008@163.com


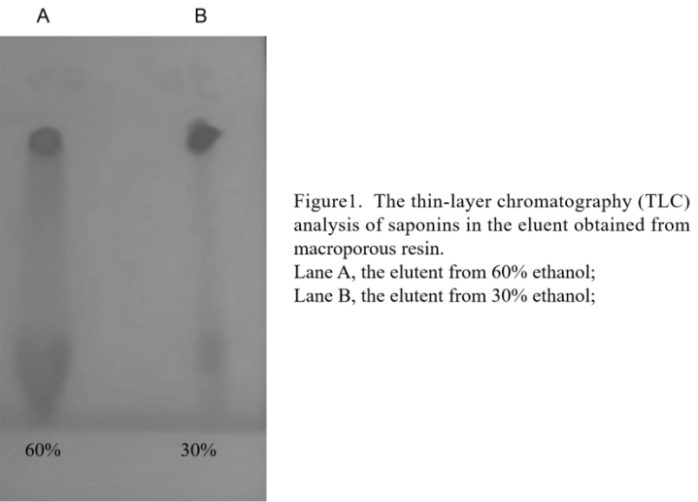


Figure S1. The thin-layer chromatography (TLC) analysis of saponins in the elutent obtained from macroporous resin. Lane A, the elutent from 60% ethanol; Lane B, the elutent from 30% ethanol.

Table S1: Analysis of constituents of SAMB by iEESI-MS


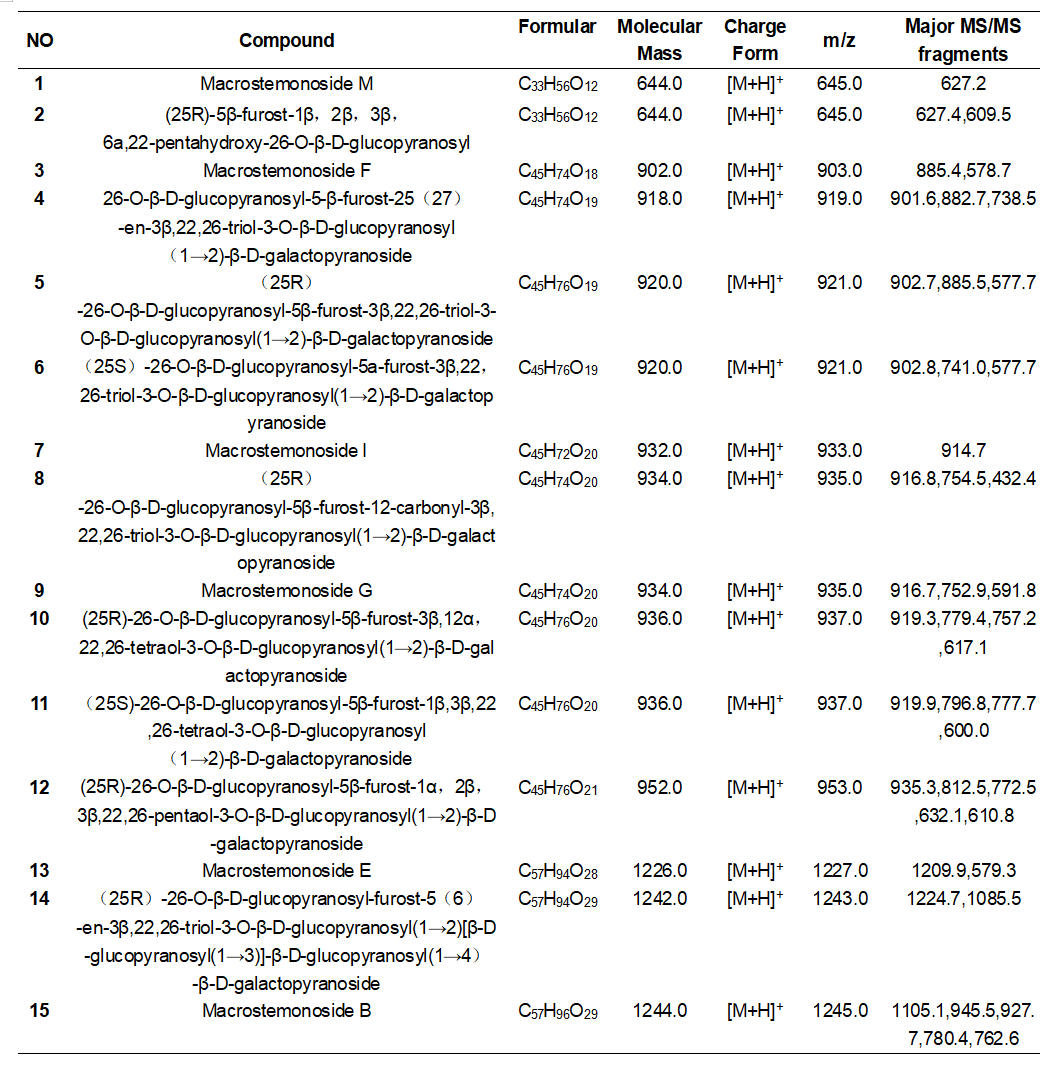


Table S2: Primers used for RT-qPCR of gene expression

| Primer | Sequence（5’-3’） |
| --- | --- |
| Abca1 | F: AAAACCGCAGACATCCTTCAG  R: CATACCGAAACTCGTTCACCC |
| Cd36 | F: ATGGGCTGTGATCGGAACTG  R: TTTGCCACGTCATCTGGGTTT |
| Abcg1 | F: TCACCCAGTTCTGCATCCTCTT  R: GCAGATGTGTCAGGACCGAGT |
| Scarb1 | F: FAAGTGGTCAACCCAAACGAG  R: ACGGTGTCGTTGTCATTGAA |
| Scaf1 | F: ACGACCCGCCACAATTCTC  R: CTGGAAGCCTTACTTGAAGGAG |
| Il-6 | F: TAGTCCTTCCTACCCCAATTTCC  R: TTGGTCCTTAGCCACTCCTTC |
| Il-1β | F: AGTTGACGGACCCCAAAAG  R: AGCTGGATGCTCTCATCAG |
| Tnf-α | F: CCCTCACACTCAGATCATCTT  R: GCTACGACGTGGGCTACAG |
| Inos | F: CTTTGCCACGGACGAGAC  R: TCATTGTACTCTGAGGGCTGAC |
| Actb | F: FTGTTACCAACTGGGACGACA  R: GGGGTGTTGAAGGTCTCAAA |


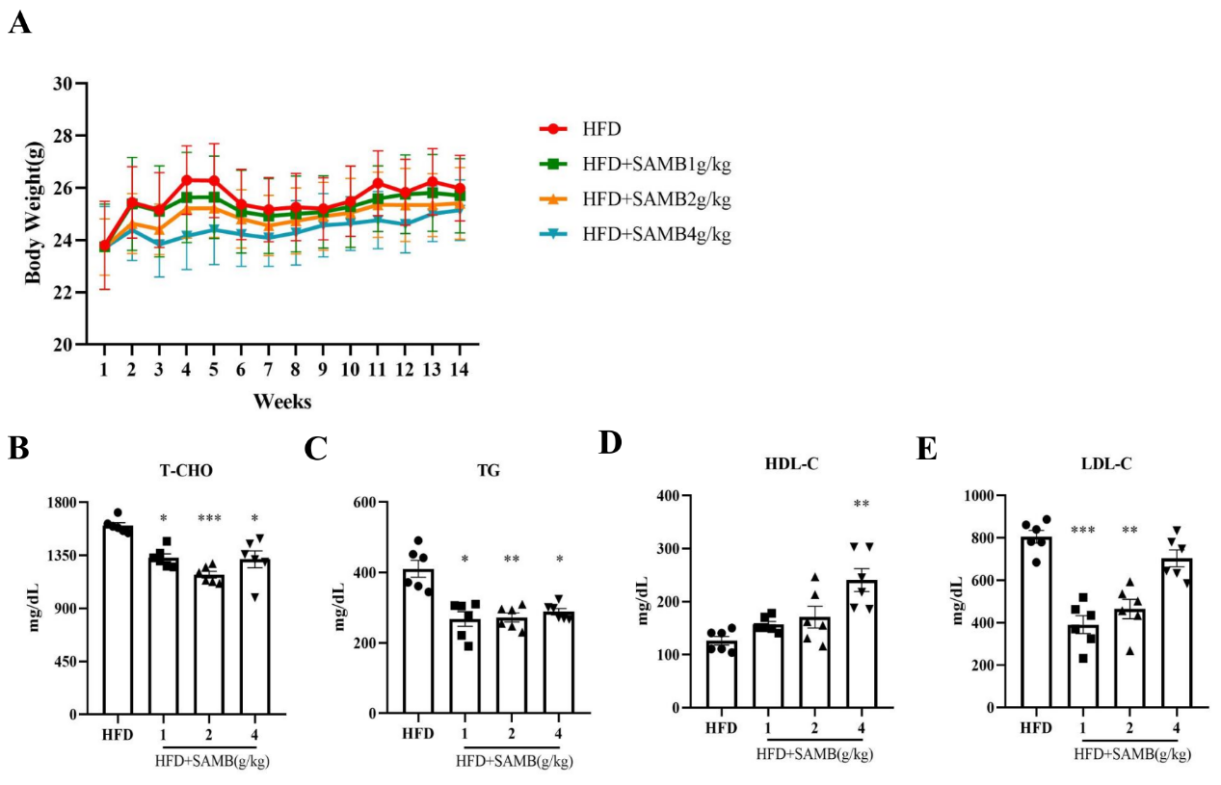


Figure S2. Quantification of weights and serum lipid concentrations in ApoE^-/-^ mice fed a high-fat diet. (A) The ApoE^-/-^ mice were subjected to a high-fat diet for 14 weeks, and their weights were measured weekly. (B-E) The concentrations of TC, TG, HDL-C, and LDL-C in the serum of each group were determined using a microplate reader according to kit instructions. Data are mean ± SEM. *P < 0.05, **P < 0.01, ***P < 0.001 vs HFD.


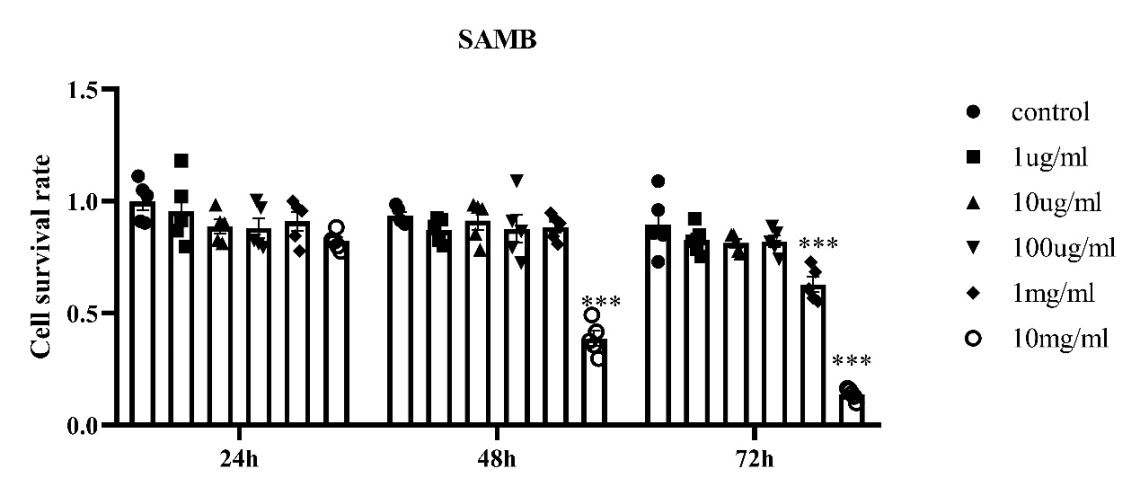


Figure S3. The cytotoxicity of SAMB on BMDM was assessed using the CCK8 assay. Data are mean ± SEM. ***P < 0.001 vs control.

**
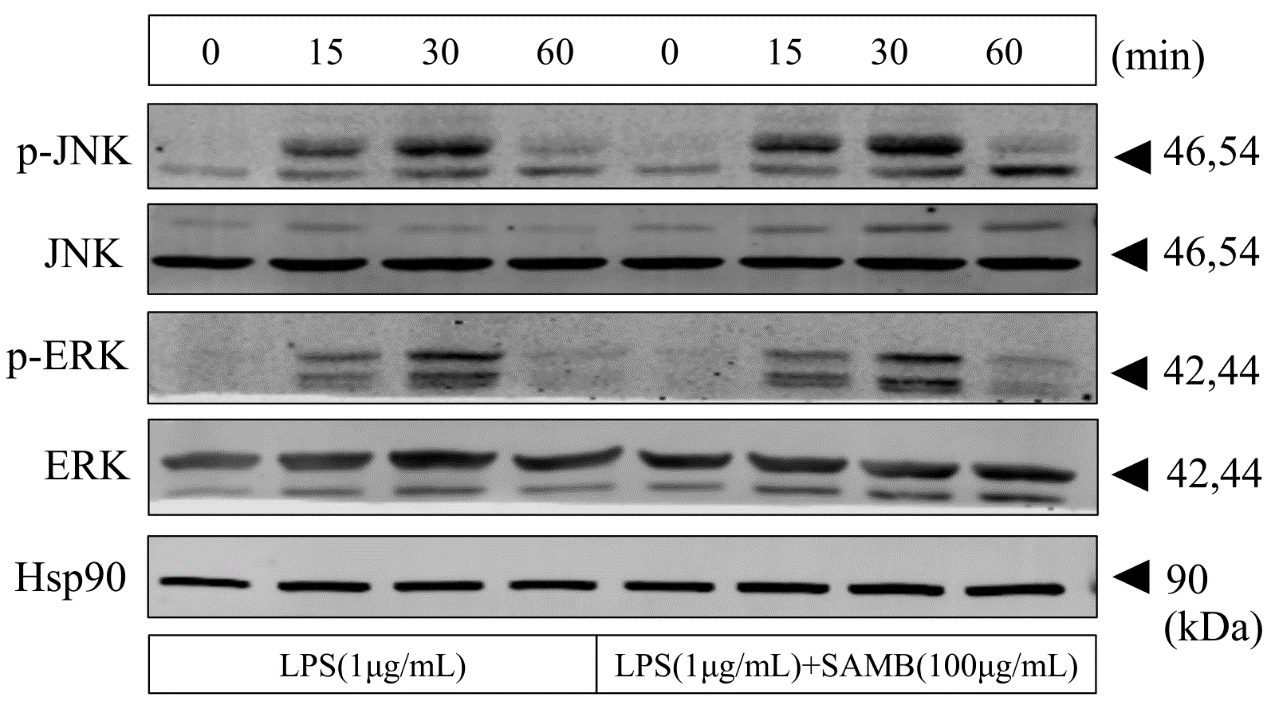
**

Figure S4. The effect of SAMB on the activation of MAPK pathways (indicated as the phosphorylation levels of JNK, and ERK ) in LPS-induced BMDMs.

**Raw data**

Fig-S1


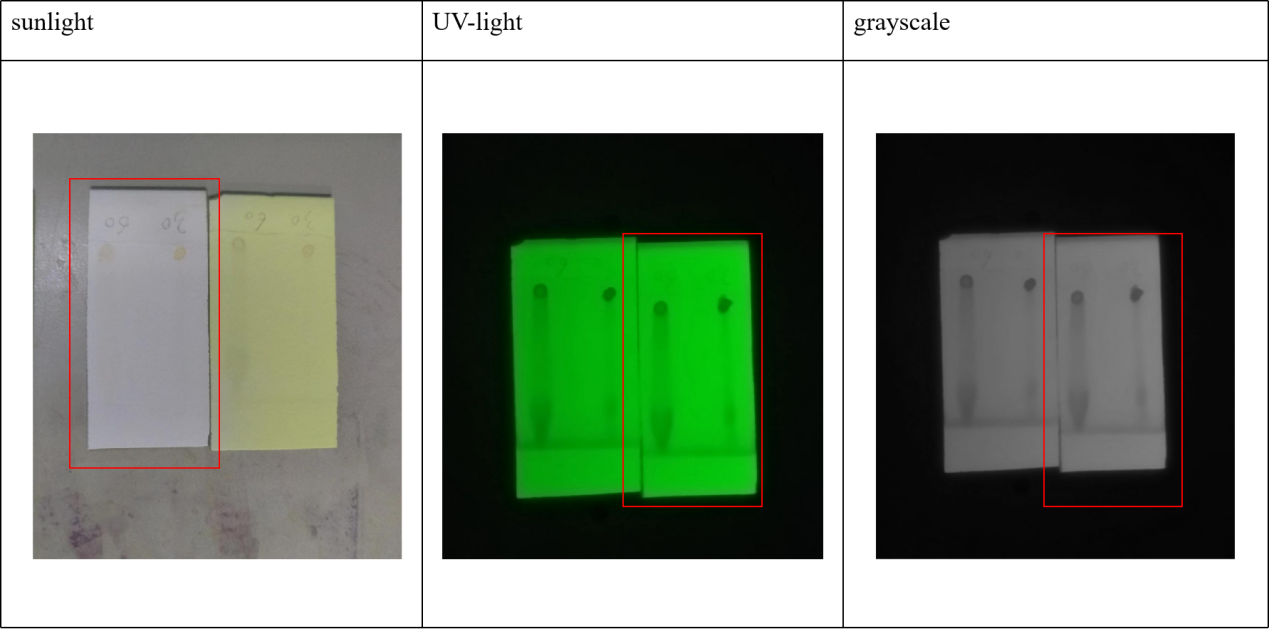


Fig-S4


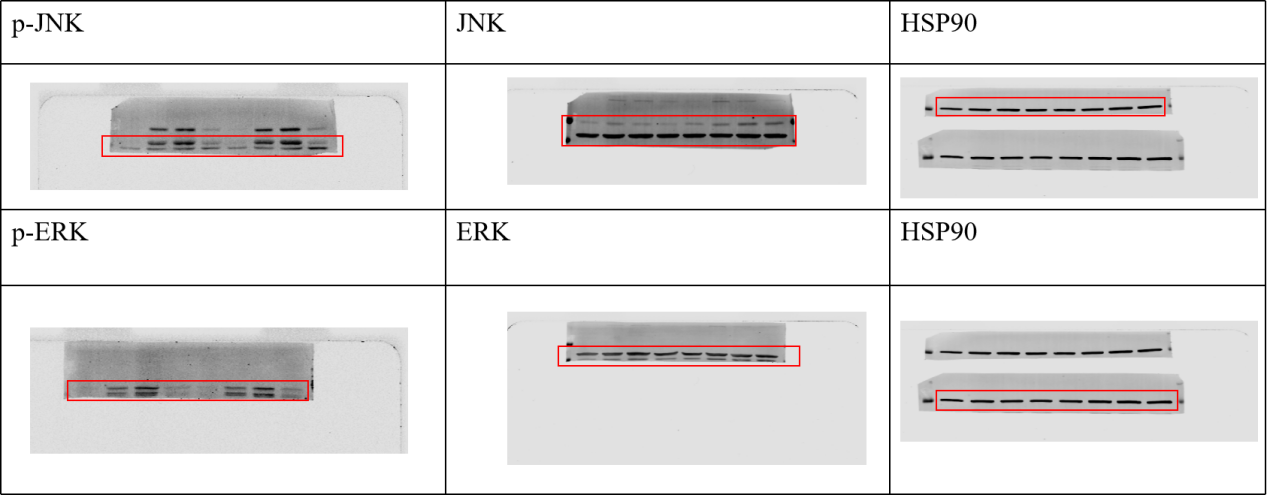


Fig-5 C


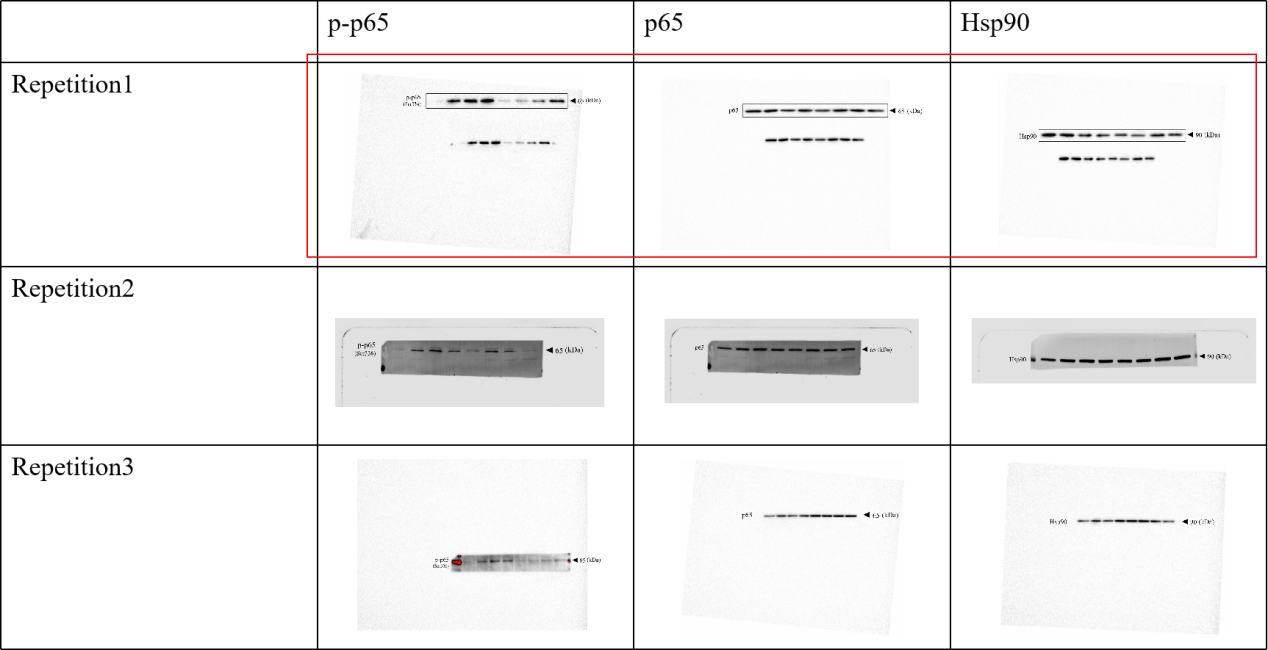


Fig-5 E


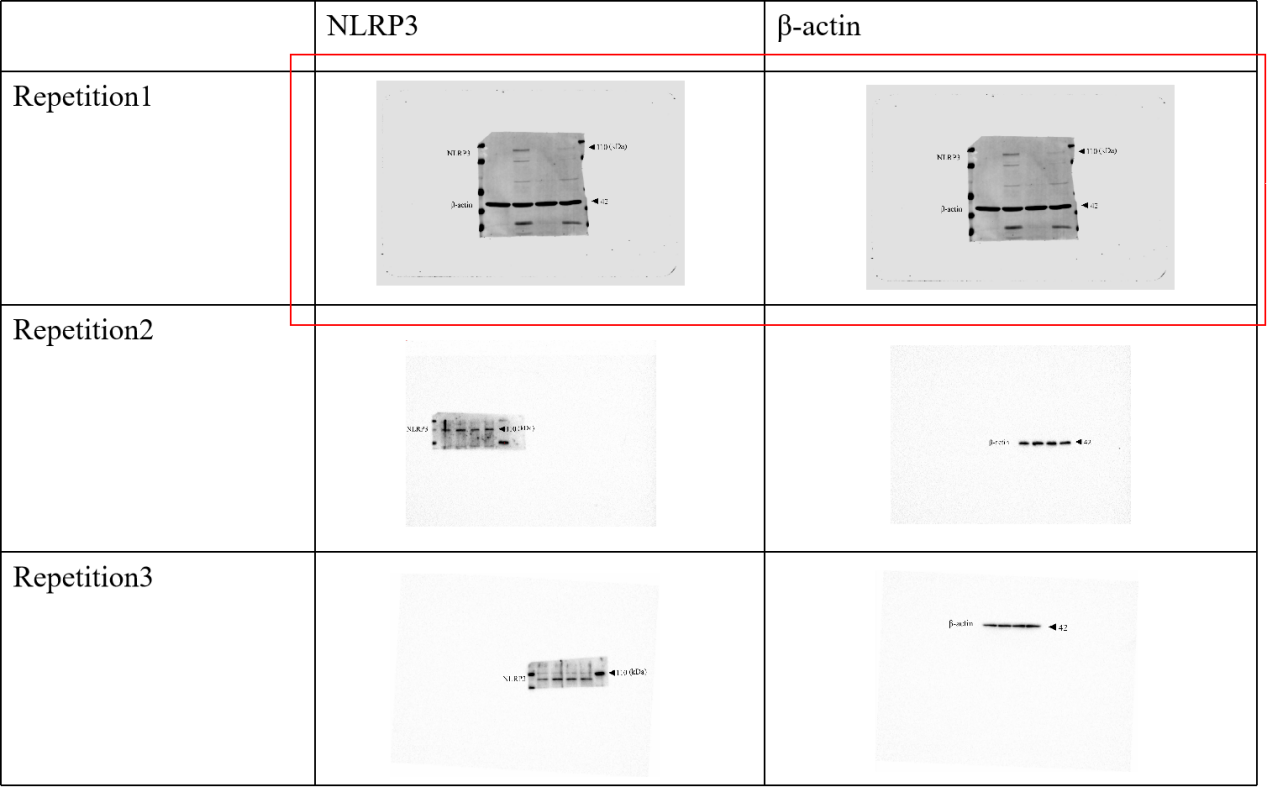


Fig-5 G


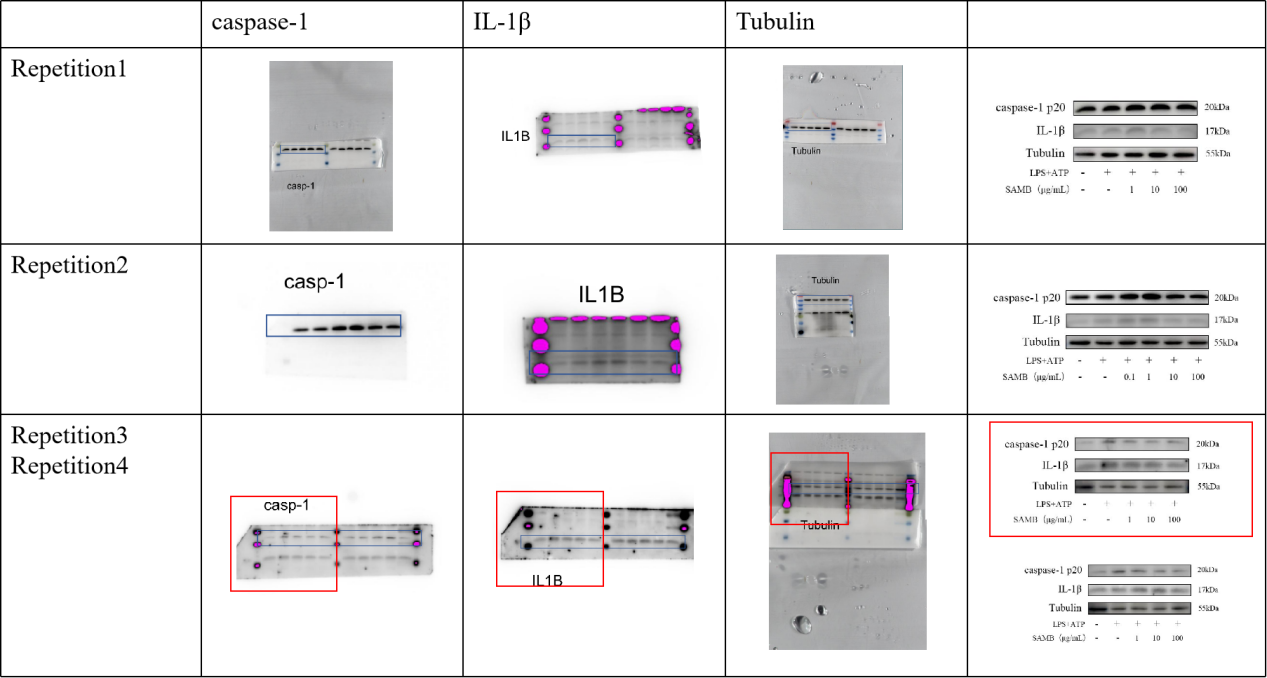

Supplement: Supplementary file 1 — Supplementary Information. [file 41598_2024_61209_MOESM1_ESM.docx]
